# How the Polls Can Be Both Spot On and Dead Wrong: Using Choice Blindness to Shift Political Attitudes and Voter Intentions

**DOI:** 10.1371/journal.pone.0060554

**Published:** 2013-04-10

**Authors:** Lars Hall, Thomas Strandberg, Philip Pärnamets, Andreas Lind, Betty Tärning, Petter Johansson

**Affiliations:** 1 Lund University Cognitive Science, Lund University, Lund, Sweden; 2 Swedish Collegium for Advanced Study, Uppsala University, Uppsala, Sweden; Universidad Carlos III de Madrid, Spain

## Abstract

Political candidates often believe they must focus their campaign efforts on a small number of swing voters open for ideological change. Based on the wisdom of opinion polls, this might seem like a good idea. But do most voters really hold their political attitudes so firmly that they are unreceptive to persuasion? We tested this premise during the most recent general election in Sweden, in which a left- and a right-wing coalition were locked in a close race. We asked our participants to state their voter intention, and presented them with a political survey of wedge issues between the two coalitions. Using a sleight-of-hand we then altered their replies to place them in the opposite political camp, and invited them to reason about their attitudes on the manipulated issues. Finally, we summarized their survey score, and asked for their voter intention again. The results showed that no more than 22% of the manipulated replies were detected, and that a full 92% of the participants accepted and endorsed our altered political survey score. Furthermore, the final voter intention question indicated that as many as 48% (±9.2%) were willing to consider a left-right coalition shift. This can be contrasted with the established polls tracking the Swedish election, which registered maximally 10% voters open for a swing. Our results indicate that political attitudes and partisan divisions can be far more flexible than what is assumed by the polls, and that people can reason about the factual issues of the campaign with considerable openness to change.

## Introduction

With the proliferation of public polls from both media, political organizations, and the parties involved, European and US elections now seems to generates almost as much controversy about the polling as the candidates and issues themselves. In particular, it has become commonplace to question the scientific integrity of the polls, and view them as partisan instruments of persuasion [1]. For example, during the recent 2012 US presidential campaign many political commentators suggested the mainstream polls were based on flawed assumptions, and harbored a systematic bias that needed to be ‘unskewed’ [2]–[4]. However, in the aftermath of the election it was concluded that professional polling organizations generally did a good job of predicting the outcome (albeit underestimating the winning margin for president Obama [5]), and that independent aggregators of the polls, such as Votamatic, FiveThirtyEight, Princeton Election Consortium, or the HuffPost Pollster was particularly accurate in their calls (see Material S1 for details).

But success in calling the outcome of a race on the eve of the election is only one aspect of the prediction game. More important in both understanding and running a campaign is the effort to delineate what *could* happen, to pinpoint how many voters are receptive to different messages, and open to ideological change. To use another example from the recent US presidential campaign; seven weeks before the election, a video was released of republican candidate Mitt Romney, secretly filmed during a fundraiser in Florida. In this video Romney declares that it is not his job not to worry about the 47% of Americans that pay no income tax, because they are not receptive to his campaign message. Instead, he asserts that there only are 5–10% of voters that are open to move across the partisan divide, and that those are the target demographic he needs to convince to win the election (for the relevant quotes, see Material S1). Independently of whether the message of the leaked tape contributed to the failure of the Romney campaign, one might legitimately ask whether it is a sound strategy to run a presidential race on the premise that maximally 10% of the electorate can be swung across party lines? Are most voters so firmly locked in their views that they are unreceptive to any attempts at persuasion, even from the concentrated effort of a billion dollar campaign machinery [6]?

Looking at the research, this seems to be the case. The most salient contrast across the political landscape in the US and the EU is the left vs. right wing division. Despite a trend towards diminishing party affiliation among voters, partisanship across the left-right divide still holds a firm grip on the international Western electorate, and has even shown evidence of further polarization in recent years (e.g. see [7]–[10] for analysis relating to the condition in the US, and [11]–[13] for the EU perspective, see also [14], [15] for cross cultural comparisons).

We were given an opportunity to test this premise during the final stretch of the 2010 general election in Sweden. Based on our previous research on the phenomenon of choice blindness (CB [16], [17]) our hypothesis was that if we could direct the focus of our participants towards the dividing policy issues of the campaign, and away from the overarching ideological labels of the competing parties, we could use CB to demonstrate far greater flexibility in their political affiliations than what is standardly assumed.

Like in the US, the Swedish electorate is regarded as one of the most securely divided populations in the world (albeit shifted somewhat to the left compared to the US continuum). When we entered into the study, the tracking polls from commercial and government institutes were polling the Swedish electorate at about 10% undecided between the two opposing coalitions social democrats/green vs. conservatives (provided by Statistics Sweden (J. Eklund, unpublished data, 2012)), with the conventional wisdom of political science identifying very few additional voters open for a swing at the final stretch of the campaign [18]–[20].

## Methods

### Participants

**Figure 1:**
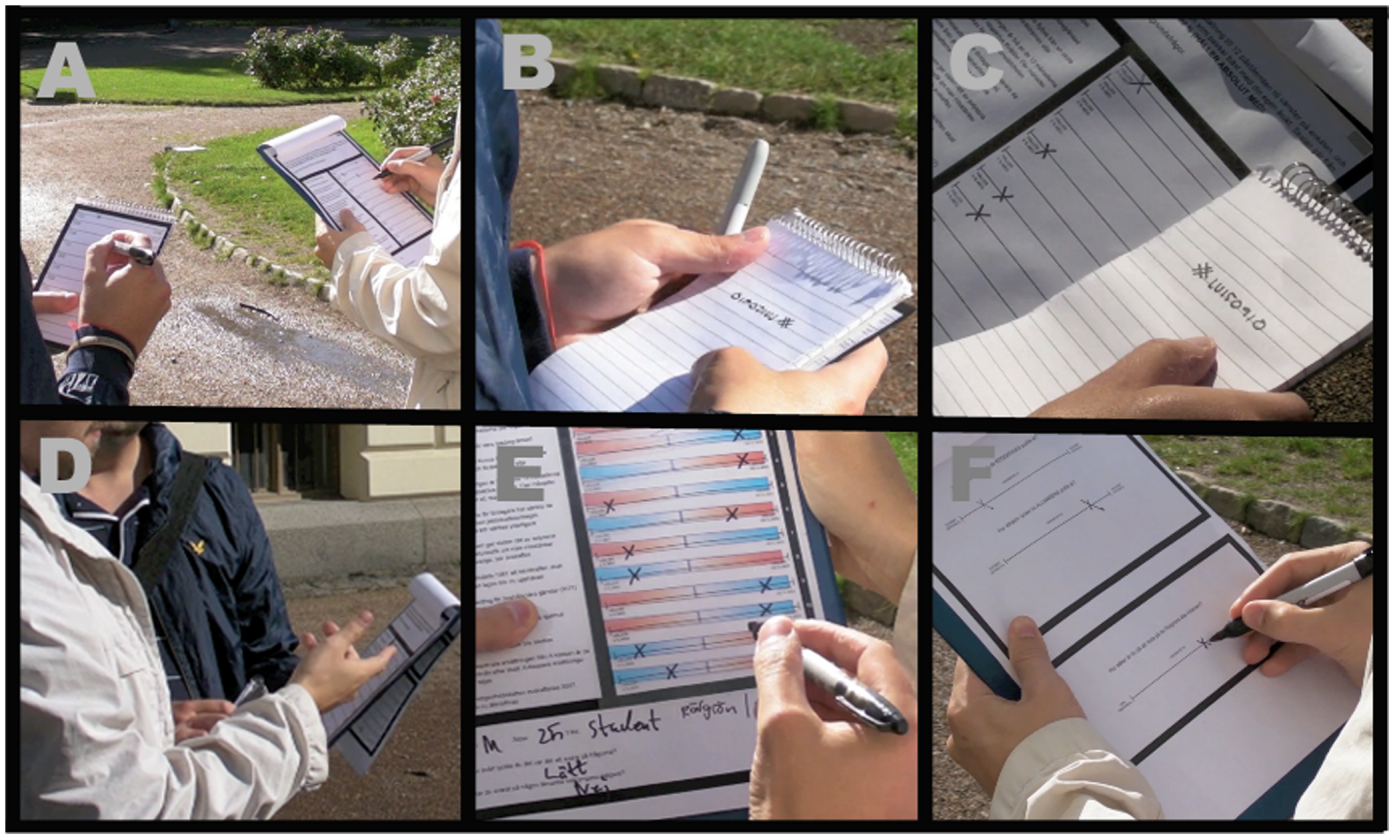
A step-by-step demonstration of the manipulation procedure. **A.** Participants indicate the direction and strength of their voting intention for the upcoming election, and rate to what extent they agree with 12 statements that differentiates between the two political coalitions. Meanwhile, the experimenter monitors the markings of the participants and creates an alternative answering profile favoring the opposite view. **B.** The experimenter hides his alternative profile under his notebook. **C.** When the participants have completed the questionnaire, they hand it back to the experimenter. The backside of the profile is prepared with an adhesive, and when the experimenter places the notebook over the questionnaire it attaches and occludes the section containing the original ratings. **D.** Next, the participants are confronted with the reversed answers, and are asked to justify the manipulated opinions. **E.** Then the experimenter adds a color-coded semi-transparent coalition template, and sums up which side the participants favor. **F.** Finally, they are asked to justify their aggregate position, and once again indicate the direction and strength of their current voting intention. See http://www.lucs.lu.se/cbp for a video illustration of the experiment.

In total, 162 volunteers (98 female) divided in two conditions (manipulated and control) participated in the study. Ages ranged from 18 to 88 years (M = 29.7, SD 14.1). We recruited our participants from various locations in the cities of Malmö and Lund in Sweden, and asked them if they wanted to fill in a questionnaire concerning their views on political issues. Participants who did not intend to vote, or who had already voted by mail were not admitted into the study. Two participants were removed due to technical problems with the manipulation process (the glued-on piece of paper did not stick and fell off during the discussion, see procedure figure 1). All participants gave informed consent.

### Ethics Statement

The study was approved by the Lund University Ethics board, D.nr. 2008–2435.

### Procedure and Materials

We introduced ourselves as researchers from Lund University with an interest in knowing the general nature of political opinions. We emphasized that participation was fully anonymous, that we had no political agenda, and that we would not argue with or judge the participants in any way. After this, we presented the participants with an ‘election compass’; a survey with salient issues from the ongoing election campaign where the left- and the right-wing coalition held opposite positions.

At the start of the questionnaire, the participants were asked to indicate how politically engaged they were (on a scale from extremely disengaged, to extremely engaged), and how certain they were in their political views (from extremely uncertain, to extremely certain). Next, they were asked to indicate the direction and certainty of their current voting intention on a 100 mm bidirectional scale (from extremely certain social democrat/green, to extremely certain conservatives, with the midpoint of the scale representing undecided).

The main survey consisted of 12 salient political issues taken from the official coalition platforms where the two sides held opposing views. On the survey, the issues were phrased as statements, such as: “*Gasoline taxes should be increased*” or “*Healthcare benefits should be time limited*”. We asked the participants to indicate their level of agreement with the statements on a 0–100% scale (where 0% meant absolutely disagree, and 100% absolutely agree, and the midpoint represented uncertainty/indecision). To avoid any obvious patterning of the answers on the form, the statements were formulated both in the positive and the negative (i.e. to introduce or to remove a particular policy) and counterbalanced for the left and right wing coalitions (see table 1).

**Table 1:** The “Election Compass” with statements describing issues that divide the two coalitions.

1. Gasoline taxes should be increased
2. Healthcare benefits should be time limited
3. It should be possible for disruptive students to be moved from a school even against the students’ and their parents’ wishes
4. Family leave benefits reserve two months out of a total of 13 months for each of the parents. The number of months that are earmarked for each parent should be increased, to insure greater equality
5. Employee income taxes have been lowered the past several years through the Earned Income Tax Credit. Income taxes should be lowered further
6. The law that gives the Swedish government the right to monitor email- and telephone traffic, if it suspects an external threat against Sweden, should be abolished
7. Sweden decided in 1997 that nuclear energy should be shut down. That law should now be repealed
8. A tax deduction for housekeeping services was established in 2007. It should be abolished
9. Running major hospitals as private establishments should be permitted
10. The legal age for criminal responsibility should be lowered
11. The maximum unemployment insurance benefit is about 11 000 Swedish Kronor per month after taxes. It should be increased
12. The wealth tax was abolished in 2007. It should be reinstated

In the neutral condition (N = 47), after having rated their agreement with the 12 statements, we asked the participants to explain and justify their stance on some of the issues. When they had completed these justifications we then overlaid a color-coded semi-transparent coalition template on their answering profile, with red indicating left-wing and blue right-wing (note, these colors are inverted in US politics). In collaboration with the participants, we then tallied an aggregate ‘compass score’ for the right and left wing side, indicating which political coalition they favored based on the policy issues presented. We then asked the participants to explain and comment on the summary score, and as the final step of the experiment, to once again indicate the direction and strength of their voting intention for the upcoming election.

However, in the manipulated condition (N = 113), while observing the participants filling out the form, we surreptitiously filled out an answer sheet identical to the one given to the participants, but created a pattern of responses supporting the opposite of their stated voting intention. Thus, if their voting intention supported the social democrat/green coalition, we made a summary compass score supporting the conservatives, and vice versa (for those that were unsure in their original voting intentions, we created an answer profile that was the opposite of their compass score). Then, before we asked the participants to discuss and justify their ratings of the individual questions, we performed a sleight-of-hand to overlay and attach our manipulated profile on top of their original answers (see Figure 1, and Material S1 for the background to the trick). Consequently, when we asked the participants to discuss their answers, they were faced with an altered position supporting the opposing coalition. For example, if they previously thought the gasoline tax ought to be raised, they were now asked to explain why they had indicated it ought to be lowered.

The goal of our alterations was to bring the sum of the participants’ answers securely to the opposing side. Thus, the number of altered responses we made on the mirrored profile depended on how directionally skewed the original answers were (say 11-1 vs. 7-5). In addition, there was no predetermined rule for the size of the manipulations across the scale. Instead, each manipulation was made with the intent of creating an overall believable pattern of responses on the profile (i.e. as the level of polarization generally varied between questions, it would invite suspicion to simply move all responses the minimal distance across the midline of the scale). During the discussion, and later during the summation, if the participants realized their answers were not expressing their original opinion, they were given the opportunity to change the rating to what they instead felt appropriate. This way, our efforts at creating a coalition shift could be nullified by the number of corrections made by the participants.

As in the neutral condition, after reacting to the summary score, the final step of the experiment was for the participants to once again indicate their voting intentions for the upcoming election.

After the experiment we explained the true purpose of the study to all participants, and demonstrated the procedure of the manipulation. At this point we asked whether they had suspected anything was wrong with their answers (over and above any previously registered corrections). We then interviewed the participants about how they felt about the experiment, and finally, everybody gave written consent to have their results included in the analysis. After the study, the experimenter took notes about the comments and explanations of the participants.

## Results

### Correction of Manipulated Answers

Each participant had on average 6.8 (SD = 1.9) answers manipulated, with a mean manipulated distance of 35.7 mm (SD = 18.7) on the 100 mm scale. The participants were explicitly asked to state reasons on average 4.0 (SD = 1.6) of the manipulated trials, and of those were on average 0.9 (SD = 1.0) answers corrected by the participants to better match their original intention (i.e. a trial-based correction rate of 22%). At an individual level, 47% of the participants did not correct any answers, while 53% corrected between 1–4 answers. For all answers classified as corrected, the participants indicated that they had misread the question, or marked the wrong end of the scale. Only a single participant expressed any suspicion that we had manipulated her profile.

The number of corrected answers were not related to gender, age, or political affiliation as defined by prior voting intention (p = n.s.). The distance being manipulated on the scale did not differ between corrected and non-corrected answers (p = n.s.). Finally, there were no differences in self-rated political engagement or in political certainty between participants who corrected no answers and participants who made one or more corrections (p = n.s.) (See Table S1 for details).

### Endorsement of Compass Score

As very few manipulated issues were corrected, we were able to create a mismatch between the initial voting intention (or original compass score for the uncertain group) and the manipulated summary score for a full 92% of the participants, all of which acknowledged and endorsed the manipulated score as their own.

### Change in Voting Intention

**Figure 2:**
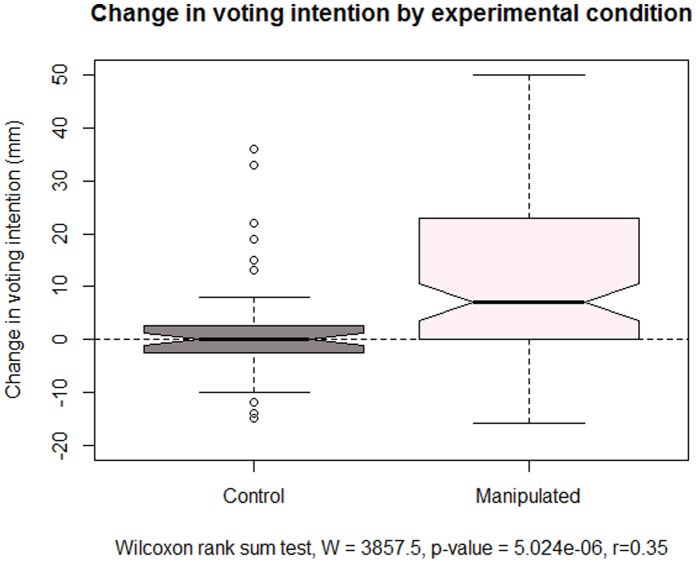
Change in voting intention in the control and in the manipulated condition.

In order to establish if the mismatch between the initial voting intention and the manipulated compass score also influenced the participants final voting intention, we measured the change in voting intention from pre- to post-test, and classified it as a positive change if it was congruent with the manipulated compass score, and as a negative change otherwise. For example, if the participants had a (manipulated) compass score biased towards the right wing, and their voting intention shifted towards the right-wing coalition, this was classified as a positive change. For the control condition, the change between initial and final voting intention was classified as positive or negative against their unaltered compass score. Using this measure to compare the amount of change in voting intention between the manipulated and the control condition, we find that there is a very large change in the manipulated condition (M = 15.9, SD = 24.7) while there is virtually no change (M = 1.72, SD = 9.9) in the control condition (Wilcoxon Rank Sum Test, W = 3857.5, p<.00001, r = 0.35, see figure 2).

In the manipulated condition, we also find that the skewness of the compass score correlates with the amount of change in voting intention, e.g. if an initially right-wing participant finds herself with a left wing aggregate score of 10 vs 2, she is likely to change her voting intention more than if the balance was 7 vs 5 (Pearson correlation, r = 0.28, p<0.005).

As was the case with level of correction, we found no connection between gender, age, level of political engagement, overall political certainty, or initial political affiliation, in relation to magnitude of change in voting intention (p = n.s.) (See Table S2 and Figure S1 for details).

If we translate the change in voting intention to categorical political affiliation, what we find is that 10% of the participants in the manipulated condition moved across the full ideological span, and switched their voting intention from firmly right wing to firmly left wing, or in the opposite direction (with a mean movement of voting intention across the scale = 71 mm, SD = 30.2). A further 19% went from expressing certain coalition support (left or right), to becoming entirely undecided (M = 27.2, SD = 13.2), and 6% went from being undecided to having a clear voting intention (M = 12.0, SD = 26.9). If we add to this the 12% that were undecided both before and after the experiment, it means that 48% (±9.2%) of the participants were willing to consider a coalition shift. In addition, a further 10% of the participants recorded substantial movement in the manipulated direction, moving 20 mm or more on the 100 mm scale.

Excluding the initially undecided participants (as they are per definition open to change), the average certainty of the initial voting intentions of the participants was notably high (M = 37.4 mm, SD = 13.45, with the 100 mm bidirectional scale transformed to a 50 mm unidirectional scale). If we compare the participants that altered their voting intention with those that did not change, we find that the latter group has a higher level of polarization (M = 34.0, SD = 14.40; M = 40.5, SD = 11.89, Wilcoxon Rank Sum Test, W = 789.5, p-value <0.05), indicating that they are somewhat more resistant to change. However, there were no differences in certainty of initial voting intentions between participants who made corrections (M = 30.0, SD = 18.58) and participants who did not make any corrections (M = 31.3, SD = 19.36)(Wilcoxon Rank Sum Test, W = 1681, p = n.s.), which indicates that greater certainty of voting intentions does not in itself translate to a greater general awareness about one’s political attitudes.

When looking at the post-experiment notes, one salient pattern we find is that around 50% of the participants who were not influenced by the manipulation referred to their ideological identity or prior voting behavior as a reason for ignoring the incongruent compass score. More generally, for all categories of participants, many also expressed clear surprise and curiosity over the fact that they failed to correct the manipulations, then argued the opposite of their original views, and finally accepted the altered compass score.

## Discussion

There are three key steps in the current result.

First, the low correction rate of the manipulated campaign issues. As reported above, the manipulations we made were generally not drastic, but constituted substantial movement on the scale, and each one of them had definitive policy implications by moving the participants across the coalition divide on issues that would be implemented or revoked at the coming term of government (yes, politicians keep most of their promises! [21], [22]). It is unlikely that the low level of corrections resulted from our use of a continuous response profile, as we observed similar results in a previous study of morality with a discrete numerical scale [17]. In fact, the survey concerned highly salient issues like income- and wealth taxation, health- and unemployment insurance, and environmental policies on gasoline and nuclear power. As such, they were both familiar and consequential, and the participants often presented knowledgeable and coherent arguments for the manipulated position (e.g. in contrast to [23], [24], who argue that voters generally lack knowledge about political facts).

Another noteworthy finding here is that we found no relationship between level of corrections and self-rated political engagement or certainty. That is, participants who rated themselves as politically engaged, or certain in their political convictions, were just as likely to fail to notice a manipulation. This complements a similar result from [17], and indicates that general self-reports of moral- or political conviction has a low sensitivity to predict correction rates on CB tasks.

The second main step of the study was the summation of the compass score. Here, an overwhelming majority of the participants accepted and endorsed a manipulated political profile that placed them in the opposite political camp. As we see it, this result is both obvious and remarkable; obvious, in that unless the participants had suspected some form of manipulation on our side, endorsement of the score follows logically from the summation (the adding was fully transparent, so it must be *their* score); and remarkable in that a few individual CB manipulations can add up to seriously challenge something as foundational as left- or right wing identity, a division seen by both academic research and commercial polling as one of the most stable constructs in the political landscape [7], [8].

But one can have many other reasons for giving political support than enthusiasm or disdain for specific policies (issues having to do with ideological commitment, trustworthiness, leadership, etc). So, the third and most critical part of the study concerned whether the participants’ endorsement of the ‘factual’ compass score would translate to a willingness to change their actual voting intentions. Here, it must be remembered that the study was conducted at the final stretch of a real election campaign, and our ratings indicated our participants were highly certain in their voting intentions from the onset. Despite this, what we found was that no less than 48% of them were being open for movement across the great partisan divide (or ‘in play’, as the pollsters would say). Adding to this the further 10% that moved more than 20 mm in the manipulated direction, often from positions at the absolute far ends of the scale, it is clear that our participants demonstrate a great deal of ideological flexibility.

This result can be compared to recent studies that have emphasized how hard it is to influence peoples’ voting intentions with ‘regular’ social psychology tools, like framing and dissonance induction [25], [26] (but see [27]). Still, most likely, our findings underestimate the number of participants open to a coalition shift. As we measured voting intentions both before and after the survey, we set up a clear incentive for the participants to be consistent across measurement (e.g. [28]–[31]). If we instead had measured voting intention only at the end of the experiment, and used the untampered compass score as a proxy for their political affiliation, they would have had no previous anchor weighing on the final voting question, and the amount of influence would probably have been larger. Similarly, our survey contained the critical wedge issues separating the coalitions, but not any party specific interests, and some participants found they could dismiss the compass score as not representative of their critical concerns (whether this was a post-hoc rationalization or not, we cannot know). However, as our result revealed there was no difference in correction rate between smaller and larger manipulations on the scale, to gain additional force for the summation score, we could have allowed the participants to indicate which issues they cared the most about, and then focused our CB manipulations there.

As argued by Haidt [32], [33], political affiliation can be seen as primarily being about emotional attachment, an almost tribal sense of belonging at the ideological level. The goal of our study was to use CB to circumvent this attachment, and get our participants to exercise their powers of reasoning (post-hoc, or not) on the factual issues of the campaign. Previous research has shown that voters engaging in ideologically motivated reasoning can be stubbornly resistant to correcting any factual misperceptions, even to the point where contradictory information presented to them only serve to strengthen their convictions [34]. Thus, in no part of the experiment did we provide arguments in support or opposition to the expressed views of the participants, instead they did all the cognitive work themselves when reasoning about the manipulated issues and the summary score. This way, it seems, we were able to peel back the bumper sticker mentality encouraged by coalition attachments, and reveal a much more nuanced stance among our participants. But nevertheless, we get a clue about the pervasive influence of ideology from what the participants reported at the end of the experiment. Particularly interesting are those participants that did not alter their voting intention. In this category, many referred to an overarching sense of coalition identity to motivate why the manipulated compass score did not influence them. Sometimes these participants even expressed a form of ideological relief at the debriefing stage (“pheeew… I’m not a social democrat after all!”).

In summary, we have demonstrated considerable levels of voter flexibility at the cusp of a national election, with almost half of our participants willing to consider a jump across the left-right divide. As the recent assessment of the polling organizations and the polling aggregators in the US confirmed, stated voting intentions in the final weeks before an election are generally very reliable [18], [19]. This was precisely the reason we chose to conduct our study at the stretch of a real campaign. But our result provides a dramatic contrast to the established polls tracking the Swedish election, which indicated that maximally 10% of the population would be open to swing their votes, or the 5–10% of uncertain voters that Mitt Romney revealed as the exclusive target of his US presidential campaign (already in May, half a year before election day). In this way, it can be seen how the polls can be spot on about what will likely happen at the vote, yet dead wrong about the true potential for change among the voters. We are happy that only five dollars’ worth of paper and glue is required to make this point, rather than a billion dollar campaign industry, but we would advise politicians against taking to the streets with a merry horde of choice blindness pollsters! Our result shows there is a world beyond ideological labels and partisan divisions, where people can approach the political issues of the campaign with considerable openness to change. Unfortunately, the question remains how to enter this world with no sleights-of-hand to pave the way.

## Supporting Information

Figure S1 **(A) Distribution of prior voting intentions and (B) distribution of post-test voting intentions.** The graphs show how the intentions become less polarized after the experiment.
(TIF)

Table S1 Non-significant tests reported in section “Correction of manipulated answers.”
(DOCX)

Table S2 Non-significant tests reported in section “Change in voting intention.”
(DOCX)

Material S1 **The Supporting Online Text-file.**
(DOCX)
