# Supplementary figures and images for: How the Polls Can Be Both Spot On and Dead Wrong: Using Choice Blindness to Shift Political Attitudes and Voter Intentions
Source: PLoS One. 2013 Apr 10;8(4):e60554. doi: 10.1371/journal.pone.0060554 (PMC3622694; doi:10.1371/journal.pone.0060554)

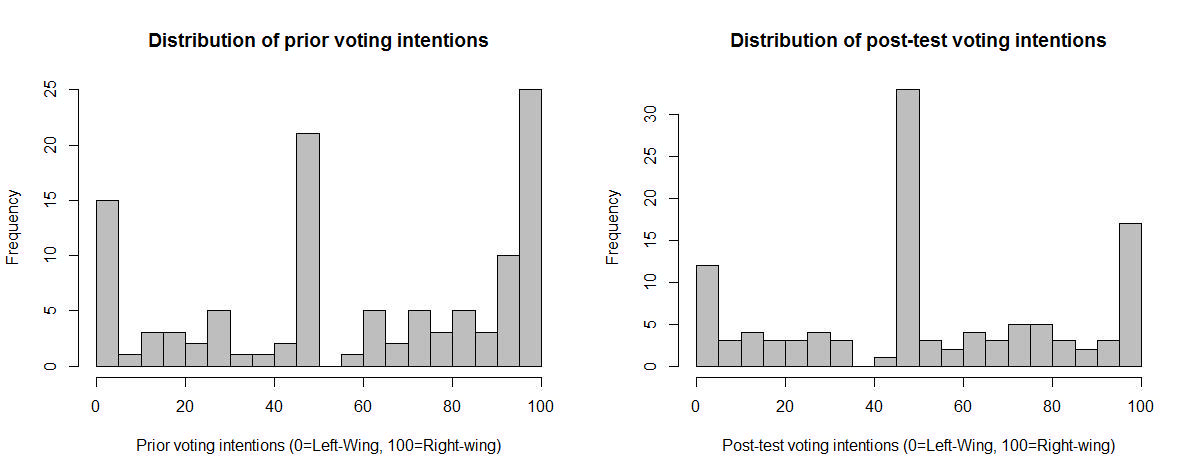

Supplement: Figure S1 — (A) Distribution of prior voting intentions and (B) distribution of post-test voting intentions. The graphs show how the intentions become less polarized after the experiment. (TIF) [file pone.0060554.s001.tif]
