# Supplementary material for: How the Polls Can Be Both Spot On and Dead Wrong: Using Choice Blindness to Shift Political Attitudes and Voter Intentions
Source: PLoS One. 2013 Apr 10;8(4):e60554. doi: 10.1371/journal.pone.0060554 (PMC3622694; doi:10.1371/journal.pone.0060554)
Supplement: Table S1 — Non-significant tests reported in section “Correction of manipulated answers.” (DOCX) [file pone.0060554.s002.docx]

Table S1.

| Variables compared | Statistical test | Descriptive + Test result |
| --- | --- | --- |
| Number of corrected answers by participant gender | Wilcoxon rank sum test with continuity correction | Males: M=.85 SD= 0.99  Females: M=.96 SD= 1.1  W = 1311, p = 0.68 |
| Number of corrected answers by participant age | Pearson's product moment correlation coefficient | r = -.05 (t(106) = -0.53, p = 0.60) |
| Making correction or not by political affiliation (left 0-45mm), neutral (45-55mm), right (55-100mm)) | Fisher’s Exact Test | Proportion correcting:  Left: 70%  Neutral: 52%  Right: 46%  p = 0.08 |
| Manipulated distance by making correction or not | Wilcoxon rank sum test with continuity correction | Correcting: M = 36.5 , SD = 10.72  Not Correcting: M = 33.6, SD = 10.68  W = 1182.5, p = 0.23 |
| Political engagement by making correction or not | Wilcoxon rank sum test with continuity correction | Correcting: M = 41.3, SD=25.50  Not Correcting: M= 42.4, SD=24.67  W = 1452, p = 0.91 |
| Political confidence by making correction or not | Wilcoxon rank sum test with continuity correction | Correcting: M = 62.3, SD=21.92  Not Correcting: M= 61.9, SD=25.88  W = 1437.5, p = 0.98 |
