# Supplementary material for: How the Polls Can Be Both Spot On and Dead Wrong: Using Choice Blindness to Shift Political Attitudes and Voter Intentions
Source: PLoS One. 2013 Apr 10;8(4):e60554. doi: 10.1371/journal.pone.0060554 (PMC3622694; doi:10.1371/journal.pone.0060554)
Supplement: Table S2 — Non-significant tests reported in section “Change in voting intention.” (DOCX) [file pone.0060554.s003.docx]

Table S2.

| Variables compared | Statistical test | Descriptives + Test result |
| --- | --- | --- |
| Change in voting intention and gender | Wilcoxon rank sum test with continuity correction | Males: M=13.2 SD= 24.22  Females: M=17.7 SD= 25.42  W = 1215, p = 0.31 |
| Change in voting intention and age | Pearson's product-moment correlation | r = -0.05, t(106)=-0.52, p=0.60 |
| Change in voting intention and political engagement | Pearson's product-moment correlation | r = - 0.04, t(106)= - 0.3932, p = 0.70 |
| Change in voting intention and political certainty | Pearson's product-moment correlation | r = - 0.03, t(106)= - 0.2828, p = 0.78 |
| Change in voting intention and prior voting intentions | Pearson's product-moment correlation | r = 0.14, t(111)= 1.5657, p = 0.12 |
