# Supplementary material for: How the Polls Can Be Both Spot On and Dead Wrong: Using Choice Blindness to Shift Political Attitudes and Voter Intentions
Source: PLoS One. 2013 Apr 10;8(4):e60554. doi: 10.1371/journal.pone.0060554 (PMC3622694; doi:10.1371/journal.pone.0060554)
Supplement: Material S1 — The Supporting Online Text-file. (DOCX) [file pone.0060554.s004.docx]

Online Supplementary Material

How the Polls Can be Both Spot On and Dead Wrong: Choice Blindness and Voter Flow Across the Partisan Divide

Lars Hall^1^, Thomas Strandberg^1^, Philip Pärnamets^1^, Andreas Lind^1^, Betty Tärning^1^ and Petter Johansson^1, 2^

^1^Lund University Cognitive Science, Lund University, Lund, Sweden

^2^Swedish Collegium for Advanced Study, Uppsala University, Uppsala, Sweden

**Independent aggregators of the polls before the 2012 US Presidential election**

Votamatic is run by Drew Linzer at <http://votamatic.org/>, FiveThirtyEight by Nate Silver at <http://fivethirtyeight.blogs.nytimes.com/>, the Princeton Election Consortium by Sam Wang at <http://election.princeton.edu/>, and the HuffPost Pollster by Simon Jackman at <http://www.huffingtonpost.com/news/pollster>. See also <http://ncpp.org/?q=node/135> for NCPP analysis of final pre-election polls and results of the 2012 US presidential election.

**Sources for the magic trick**

In developing the methodology used we were inspired by a well-known magic procedure known as the Out-To-Lunch Principle (OTL). The OTL name comes from a business card trick first marketed by Clare Cummings and Bob Ellis in Linking Ring Magazine in 1947 (see <http://www.geniimagazine.com/magicpedia/Out-to-Lunch>), but the history of the procedure dates back much further than this, and a primitive version of the OTL was published as early as 1694 by Eberhard Welper in *Das Zeitkürzende Lust- und Spielhaus*. At this time, no props were used, and in the trick the edge of a half-card on top of a deck was concealed only by the magician’s fingers.

However, even though we were inspired by OTL in developing the magic survey, the technique of hiding a covering section by using a drawn border to conceal the edges has its own history. The idea of ’framed masking’ goes back at least to séance gaffs of the 19th century, for example as seen in "The Interrupted Flap" in *Spirit Slate Writing and Kindred Phenomena* by William Robinson, 1893, in which a chalk line hides the edge. The concept have then been used on and off by magicians over the last century. For a notable modern example, see Max Maven’s "The Spirit is Willing (To Write)" in *The Blue Book of Mentalism* 1976.

**Extended Result Section**

For clarity, description of results and tests used in non-significant comparisons were omitted from the main article. These comparisons are detailed below in table S1 and table S1. Figure S1 describes the left- and right-wing distribution of prior and post-test voting intentions. In addition, a detailed comparison between the basic properties of the control and the manipulated condition is included to show that any differences between them found for dependent variables are due to differences in the experimental procedure and not to underlying differences in group properties. Finally, we have included a transcript and source for the Mitt Romney recording cited in the introduction of the main article.

**Comparison of the control and the manipulated condition**

The strength of the initial voting intention and the participants’ compass score was highly correlated in the control condition (r = 0.72 (t(45) = 6.94, p < 0.0001). We also get the same level of correlation between the participants initial voting intention and the participants’ non-manipulated compass score in the manipulated condition (r = 0.74 (t(111) = 11.52 p < 0.0001). This indicates, both that the participants are aware of the two coalition's positions on the issues presented on the political compass, and that there is an agreement between their own overall opinion and their opinions on the specific issues. The near identical levels of correlation in the control and in the manipulated condition also show that the participants in the two conditions are very similar in terms of underlying political awareness. The similarity between the control and the manipulated condition is further reinforced by the fact that there were no differences between them when comparing the self-rated level of political engagement (control condition M=39.6, SD=23.68; manipulated condition M=41.8, SD=24.92; W = 2619.5, p = 0.75) as well as the self-rated level of confidence in political issues (control condition M = 61.6, SD = 28.11; manipulated condition M = 62.1, SD = 23.51; W = 2422, p = 0.65).

Looking at the strength of the initial voting intention, with the 100 mm bidirectional scale transformed to a 50mm unidirectional scale, we again find no difference between the control (M = 39.4mm, SD = 11.89) and the manipulated condition (M=37.4mm, SD=13.45) (W = 1695, p = 0.62). Finally, there are no differences in gender distribution in the two groups (control group 63% female; manipulated group female 62%; Fisher’s Exact Test, p = 0.86) or differences in age (control condition M=31.5 , SD=17.10; manipulated condition M=29.1 , SD=12.86; W = 2343, p = 0.58)

Given the overall similarities between the control and the manipulated condition, the changes reported in the main text between pre- and post-test voting intentions for the manipulated group, and the difference between the control and the manipulated condition with respect to pre- and post-test changes in voting intention, cannot be explained as an artefact arising from underlying differences between the two groups.

**Transcripts from Mitt Romney fundraiser**

"And so when you do polls, and you ask people what is the biggest issue in the 2012 election, No. 1 is the economy and jobs by a wide margin. But No. 2 is the deficit. But debt, that doesn't calculate for folks, but the deficit does. They recognize you can't go on forever like this. Although the people who recognize that tend to be Republicans, and the people who don't recognize that tend to be Democrats. ***And what we have to get is that 5 or 10 percent in the middle who sometimes vote Republican, sometimes vote Democrat***, and have them understand how important this is"

"And I mean, the president starts off with 48, 49, 48—he starts off with a huge number. These are people who pay no income tax. Forty-seven percent of Americans pay no income tax. So our message of low taxes doesn't connect. And he'll be out there talking about tax cuts for the rich. I mean that's what they sell every four years. And so my job is not to worry about those people—I'll never convince them that they should take personal responsibility and care for their lives. ***What I have to do is convince the 5 to 10 percent in the center that are independents that are thoughtful, that look at voting one way or the other*** depending upon in some cases emotion, whether they like the guy or not"

"We speak with voters across the country about their perceptions. ***Those people I told you, the 5 to 6 or 7 percent that we have to bring onto our side***, they all voted for Barack Obama four years ago /.../ Those people that we that have to get, they want to think they did the right thing but he just wasn't up to the task. They love the phrase, "He's in over his head."

Quotes from Mitt Romney retrieved from:

http://www.motherjones.com/politics/2012/09/full-transcript-mitt-romney-secret-video

All emphasis above is ours.

**Prior Work**

This study was mentioned as work in preparation in a non-peer reviewed book chapter: Johansson P, Hall L, Chater N (2012) Preference Change Through Choice. In Sharot T, Dolan R, editors. Neuroscience of Preference and Choice. Elsevier
